# Comparison of direct measurement of intracranial pressures and presumptive clinical and magnetic resonance imaging indicators of intracranial hypertension in dogs with brain tumors

**DOI:** 10.1111/jvim.15802

**Published:** 2020-05-16

**Authors:** Savannah Giannasi, Yukitaka Kani, Fang‐Chi Hsu, John H. Rossmeisl

**Affiliations:** ^1^ Veterinary and Comparative Neuro‐Oncology Laboratory Virginia‐Maryland College of Veterinary Medicine, Virginia Tech Blacksburg Virginia USA; ^2^ Department of Biostatistics and Data Science, Comprehensive Cancer Center and Brain Tumor Center of Excellence Wake Forest School of Medicine Winston‐Salem North Carolina USA; ^3^ Department of Cancer Biology, Comprehensive Cancer Center and Brain Tumor Center of Excellence Wake Forest School of Medicine Winston‐Salem North Carolina USA

**Keywords:** brain tumor, canine, imaging biomarkers, intracranial hypertension, modified Glasgow coma scale

## Abstract

**Background:**

Intracranial hypertension (ICH) is often presumptively diagnosed based on clinical or imaging findings. Clinical or imaging surrogates of ICH are not usually validated with reference standard direct intracranial pressure (dICP) recordings.

**Hypotheses:**

Dogs with brain magnetic resonance imaging (MRI) or clinical features of presumed ICH would have higher dICP than dogs lacking those features.

**Animals:**

Twenty dogs with gliomas and 3 normal controls.

**Methods:**

Prospective, convenience study. Dogs were presumptively categorized with normal ICP or ICH from scores generated from described clinical and brain MRI indicators of ICH. dICP was recorded in anesthetized dogs using an intraparenchymal microsensor and compared between groups.

**Results:**

dICP was not different between control (10.4 ± 2.1 mm Hg) and dogs with glioma (15.6 ± 8.3 mm Hg), or between dogs in clinically predicted ICP groups. Compared with dogs with MRI‐predicted normal ICP, MRI‐predicted ICH dogs had higher dICP (10.3 ± 4.1 versus 19.2 ± 7.9 mm Hg, *P* = .004), larger tumors (1.45 ± 1.2 versus 5.71 ± 3.03 cm^3^, *P* = .0004), larger optic nerve sheath diameters, and 14/14 (100%) displayed structural anatomical shifts on MRI. At a dICP threshold of 15 mm Hg, the sensitivity of MRI for predicting ICH was 90% and the specificity 69%.

**Conclusions and Clinical Relevance:**

dICP measurements are feasible in dogs with brain tumors. MRI features including brain herniations, mass effect, and optic nerve size aid in the identification of dogs with ICH. Clinical estimation of ICP did not discriminate between dogs with and without ICH.

**Abbreviations**
AUC: area under curve
CCS: composite clinical score
CPP: cerebral perfusion pressure
dICP: direct intracranial pressure measurement
ICH: intracranial hypertension
ICP: intracranial pressure
KPS: Karnofsky performance score
MAP: mean arterial pressure
MGCS: modified Glasgow coma scale score
MRI: magnetic resonance imaging
ONSD: optic nerve sheath diameter
PGP: probe guide pedestal
TBI: traumatic brain injury
TCD: transcranial Doppler ultrasonography

## 1 INTRODUCTION

The intracranial pressure (ICP) is the pressure exerted between the calvarium and the intracranial tissues, namely the brain parenchyma, cerebrospinal fluid, and blood.^1^ An above normal the normal range is referred to as intracranial hypertension (ICH).^1^ As the brain is contained within the closed calvarium, once regulatory mechanisms that maintain normal ICP are overwhelmed, even small changes in the volume of the intracranial contents, often because of the presence of brain edema, hemorrhage, or mass lesions, can cause exponential rises in ICP.^1^ Intracranial hypertension is a common sequelae of a variety of brain diseases that can result in catastrophic neurological dysfunction or death.^1^, ^2^

Initiation of treatment based on target or threshold quantitative patient physiological data is emerging as the standard of care in humans with ICH because of traumatic brain injury (TBI), and has also been evaluated in small animals with TBI.^3^, ^4^ The presence of ICH upon anesthetic recovery has also been implicated as a negative prognostic indicator in dogs undergoing surgical treatment of intracranial neoplasia.^5^ Quantitative goal‐directed therapeutic approaches or prognostication requires the direct monitoring of ICP (dICP) along with other vital patient variables that contribute to cerebral physiology and metabolism.^3^, ^5^, ^6^ There are several instruments capable of dICP measurement, including external strain transducers, catheter tip strain‐gauge, solid state capacitance catheters, and fiberoptic catheter tip technologies.^6^ Intraventricular catheters hydraulically coupled to external strain transducers are considered the superior reference standard method for ICP measurement in humans, although all currently available systems are sufficiently safe, reliable, and accurate for clinical use.^6^

In dogs, all of these techniques can accurately measure dICP. ^2^, ^4^, ^5^, ^6^, ^7^, ^8^, ^9^, ^10^ To date, the majority of studies that have investigated dICP in dogs have used healthy dogs or those with experimentally induced ICH, with only 3 studies describing fiberoptic ICP monitoring in dogs with naturally occurring intracranial diseases, which included 22 dogs with brain mass lesions and 1 dog with TBI.^2^, ^4^, ^5^ Disadvantages associated with fiberoptic ICP systems, particularly for indications requiring chronic monitoring in animals, include the high‐profile cranial bolt patient interface, and the relatively fragile fiberoptic monitoring cable.^2^, ^9^ The feasibility, flexibility, and technical benefits of a catheter tip strain‐gauge ICP system in anesthetized and conscious dogs has been previously illustrated, which might facilitate the use of this technique in clinical practice.^10^

Because of the invasiveness of dICP methods, noninvasive indirect techniques to estimate ICP have also been applied in dogs with intracranial disease, including brain magnetic resonance imaging (MRI) features, transcranial Doppler (TCD) ultrasonography, or optic nerve sheath diameter (ONSD) quantification.^11^, ^12^, ^13^, ^14^ In experimental canine models of ICH, MRI characteristics, TCD, and ONSD are reliable surrogates of ICP when assessed in parallel with dICP measurements.^15^, ^16^, ^17^ However, studies investigating clinical and MRI features, TCD, and ONSD estimates of ICP in dogs with spontaneous intracranial disease have been limited by a lack of validation of indirectly estimated ICP data with dICP recordings.^12^, ^13^, ^14^

The objectives of this study were to: (a) evaluate the feasibility of dICP monitoring with a catheter strain‐gauge microsensor transducer in dogs with brain tumors, (b) determine if dICP values were different between dogs with brain tumors and controls, and (c) determine if indirect MRI or clinical measures of presumed ICH could discriminate dogs with and without ICH. We hypothesized that dogs with tumors would have higher dICP values than normal dogs, and dogs with MRI and clinical features of presumed ICH would have dICP values that were higher than dogs without those features.

## 2 MATERIALS AND METHODS

### 2.1 Study design and animals

**FIGURE 1:**
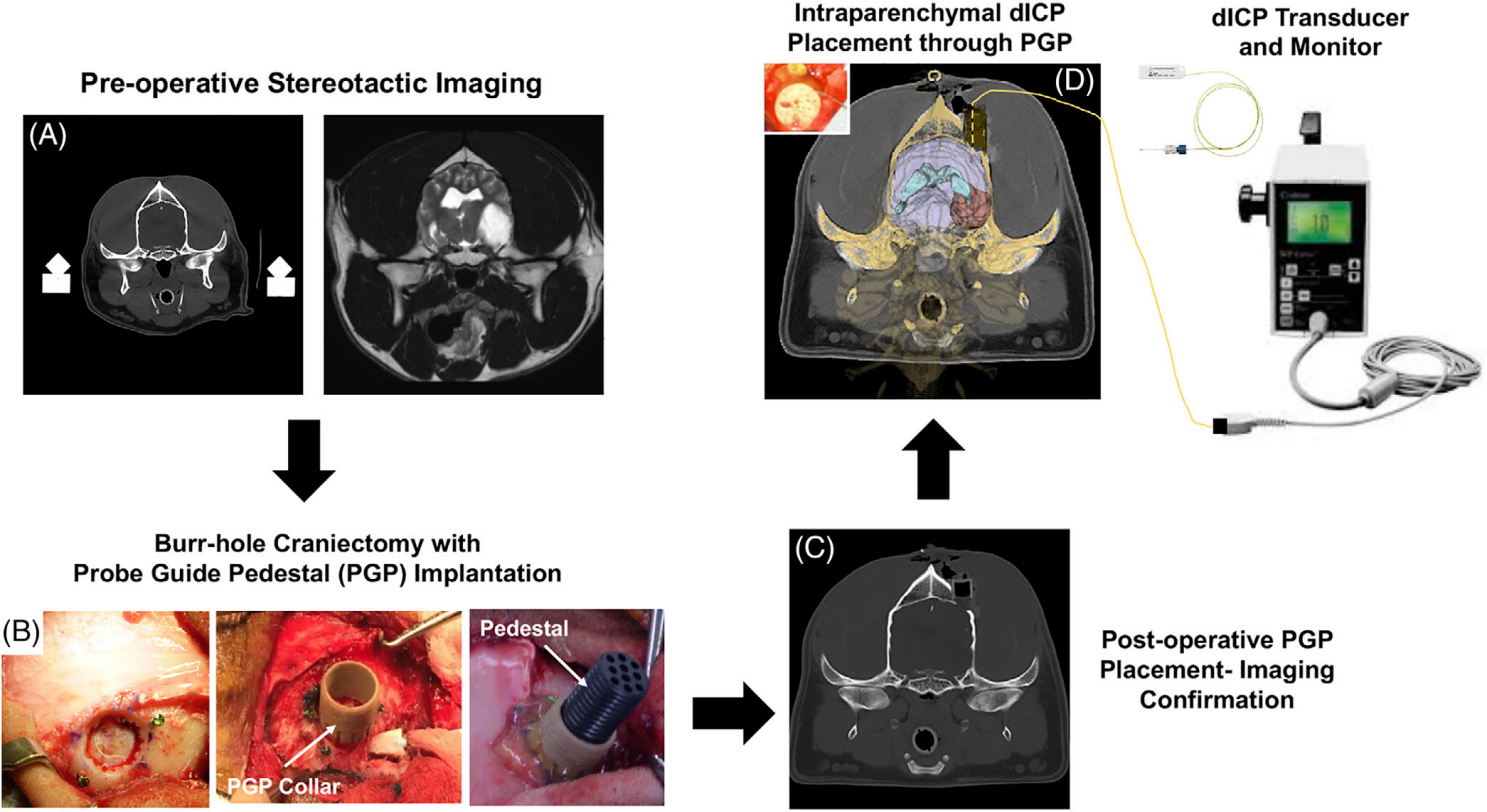
Methodological summary of direct intracranial pressure monitoring (dICP) procedure with intraparenchymal catheter strain‐gauge microtransducer. Computed tomography and magnetic resonance imaging examinations of the brain are obtained with the dog instrumented in a stereotactic headframe, A, to allow for planning for an approach to the brain and tumor that will simultaneously allow for dICP, stereotactic needle biopsy of the tumor, and catheter or electrode‐based tumor treatment through a minimally invasive burr‐hole craniectomy with probe guide pedestal (PGP) placement, B. PGP placement is confirmed with postoperative imaging, C, before treatment. The dICP transducer, D, is placed through 1 operating channel of the PGP (inset) to a depth of 10 mm into grossly normal appearing brain parenchyma, and then connected to the dICP monitor

This was a prospective, convenience sample study that included 20 consecutive dogs with histopathologically confirmed rostrotentorial gliomas that were scheduled for intraoperative dICP monitoring through a probe guide pedestal (PGP; Figure 1) implanted in the calvarium as part of clinical trials investigating catheter or probe‐based intraparenchymal therapeutic deliveries in the brain.^18^, ^19^, ^20^ Three clinically normal, purpose‐bred research dogs were included as controls.

### 2.2 Clinical scoring

Each dog had physical and neurological examinations performed by a board‐certified neurologist on the day of the dICP monitoring procedure. From the clinical examination data, dogs were assigned a fundoscopic score, Karnofsky performance score (KPS), and modified Glasgow coma scale score (MGCS) as described previously.^18^, ^19^, ^20^, ^21^, ^22^ A composite clinical score (CCS; Table 1), calculated from fundoscopic, KPS, and MCGS scores, was assigned to each dog, and CCS ≥4 was considered indicative of presumptive ICH.

**TABLE 1:** Composite clinical scoring system used to estimate intracranial pressure in dogs

Clinical examination	Score range	Adjusted score
Fundoscopic score (FS)^12^	0 = Normal 1 = Papilledema	0 or 1
Karnofsky performance score (KPS)^21^	0‐100	100‐dogscore/10 = aKPS
Modified Glasgow coma scale score (MGCS)^18^, ^22^	3‐18	18‐dog score = aMGCS
Composite clinical score (CCS)	0‐26^a^	CCS = FS + aKPS + aMGCS

a Higher CCS indicative of more severe clinical dysfunction; CCS ≥ 4 considered indicative of presumptive intracranial hypertension.

### 2.3 Brain MRI

Magnetic resonance imaging examinations of the brain were obtained under anesthesia in using a 1.5T system (Philips Intera, Andover, Massachusetts) in all dogs 48‐96 hours before the dICP monitoring procedure. All dogs were positioned in sternal recumbency and were immobilized in a stereotactic headframe during imaging examinations.^20^ Using a standardized protocol, pre‐ and postcontrast 3DT1W, T2W, T2*GRE, diffusion tensor imaging, and FLAIR sequences were obtained from all dogs.^23^ MRI features previously identified as potential indicators of ICH, including effacement of the cerebral sulci, brain herniations (foramen magnum, transtentorial, and subfalcine), compression of the 3rd or 4th ventricles, presence of perilesional edema, falx shift ≥3 mm, and displacement of the lamina quadrigemina were recorded independently by 3 investigators blinded to the dICP measurement data.^12^, ^13^, ^14^, ^15^, ^16^, ^17^ In cases of interobserver discordance, consensus was used for the final assignment of an MRI feature as present or absent. Dogs were considered to have presumptive ICH if ≥3 of these MRI findings were identified, as 19/20 tumor‐bearing dogs had at least 2 previously described MRI features of presumptive ICH present. Using reported morphometric techniques, the left and right ONSD were quantified from transverse T2W images, ONSD : body weight ratios calculated,^13^ and the lesion volume, total brain volume, and lesion : brain volume ratios determined from 2‐planar T2W region of interest segmentation with manual masking.^24^ The reported quantitative MRI variables represent the means (±SD) calculated from the 3 observers.

### 2.4 Surgical and dICP monitoring procedures

Dogs were premedicated with midazolam (0.05‐0.1 mg/kg IV) and butorphanol tartrate (0.2 mg/kg IV). General anesthesia was induced with propofol (4‐6 mg/kg IV), endotracheal intubation performed, and each dog mechanically ventilated to maintain normoxia and normocapnia (Datascope Passport 2, Mindray, Mahweh, New Jersey). Anesthesia was maintained with propofol (0.15‐0.6 mg/kg/min) and fentanyl (5‐20 mg/kg/hr) or remifentanil (0.2‐0.5 mg/kg/min) constant rate IV infusions. A catheter was placed in the dorsal pedal artery of each dog to allow for continuous direct arterial blood pressure monitoring (Datascope), and the mean arterial pressure (MAP) was maintained ≥60 mm Hg. Each dog received IV lactated Ringer's solution throughout the duration of the procedure (10 mL/kg/h).

With the dogs in sternal recumbency and instrumented in a stereotactic headframe, a unilateral rostrotentorial approach to the skull was made in each dog in order to expose the PGP collar that was implanted within a previously created burr‐hole craniectomy performed for the purpose of stereotactic tumor biopsy and subsequent catheter or probe‐based tumor treatment (Figure 1).^18^, ^19^, ^20^ In all tumor cases, the PGP was placed ipsilateral to the side of the brain mass. The PGP pedestal was threaded into the collar in preparation for passage of the dICP transducer (Codman Microsensor, Codman & Shurtleff Inc, Raynham, Massachusetts), which consisted of a miniature strain‐gauge pressure sensor mounted in the end of a 1‐m, flexible monitoring cable that connects to the control monitor (Codman ICP Express, Codman & Shurtleff Inc).^5^, ^10^ Before dICP transducer placement, transducer absolute error was verified (≤1 mm Hg) by measuring pressure within a distilled water column, and the transducers were zeroed and calibrated in a vessel of sterile 0.9% NaCl solution according to the manufacturer's instructions.^10^ In the control dogs, dICP measurements were obtained from the postcruciate gyri of the cerebrum. The location of rostrotentorial intraparenchymal dICP transducer placement within the cerebrum was not standardized in dogs with gliomas. To avoid intratumoral dICP placement in dogs with large tumor burdens or tumors that contacted the meninges, PGP were implanted such that each dog had at least 1 operating channel trajectory that allowed placement of the dICP transducer into brain tissue that appeared macroscopically normal on MRI. After puncture of the dura with a perforator (Durapierce, Codman & Shurtleff Inc), dICP transducers were passed through a Tuohy peel‐away introducer (Wiley Spinal, Epimed, Dallas, Texas) placed in operating channel of the PGP to a depth of 10 mm into the cerebral parenchyma, and then the introducer was removed (Figure 1).

After transducer placement, manual compression of the jugular vein was performed to evaluate in situ dICP transducer performance, which resulted in a 4‐20 mm Hg increase in ICP within 30‐45 seconds. Jugular compression was then released and after a 5‐minute equilibration period, the MAP recorded every 5 minutes, and the dICP recorded every minute for 15 minutes, before the initiation of other planned surgical interventions. The cerebral perfusion pressure (CPP) was subsequently calculated at 5‐minute intervals from MAP and dICP data (CPP = MAP‐dICP). Only physiological recordings that were made or calculated from the 15 minutes before the onset of the therapeutic surgical procedure were included in data analyses.

### 2.5 Statistical analyses

Means, standard deviations, medians, and ranges were calculated for continuous characteristics. Counts and proportions were calculated for discrete characteristics. Two‐sample*t* tests were used to compare KPS, MGCS, and CCS scores between control and tumor dogs, between dogs with clinically predicted ICH and those with clinically predicted normal ICP, and between dogs with MRI‐predicted ICH and those with MRI‐predicted normal ICP. Mixed effects models (PROC MIXED in SAS) were used to examine the association between ICP, MAP, and CPP as well as clinically predicted ICH groups (ICH versus normal ICP), MRI‐predicted ICH groups (ICH versus normal ICP), and disease status (tumor versus control) over time. ICP, MAP, and CPP were the outcome variables and their distributions were checked in order to satisfy the normality assumption. We accounted for the correlated repeated measures over time using a random intercept and an unstructured covariance matrix.

Interobserver agreement between the 3 raters was determined for the qualitative binary (present versus absent) MRI features including sulci effacement, transtentorial herniation, foramen magnum herniation, subfalcine herniation, lamina quadrigemina position, perilesional edema, 3rd ventricular compression, 4th ventricular compression, falx shift, and MRI‐predicted ICH by calculating pairwise Kappa agreement statistics.

Fisher exact tests were used to compare the proportions of binary MRI features including sulci effacement, transtentorial herniation, foramen magnum herniation, subfalcine herniation, lamina quadrigemina position, perilesional edema, 3rd ventricular compression, 4th ventricular compression, and falx shift between the 2 MRI‐predicted ICP groups. Two‐sample*t* tests were used to compare quantitative MRI features that were normally distributed, including T2W lesion volume, T2 brain volume, T2W lesion volume : brain volume, and right and left ONSD : body weight, between MRI‐predicted ICP groups.

Area under the curve (AUC) statistics of the receiver operating characteristic curve were used to assess the prediction performance of clinical and MRI‐predicted ICP groups to discriminate dogs with and without ICH defined by dichotomous dICP thresholds of ≥15, ≥20, and ≥25 mm Hg. AUC quantifies the overall ability to discriminate between those who have the ICH and those who do not, and ranges from 0.5 (useless) to 1 (perfect). AUC and its confidence interval were estimated. Sensitivity and specificity were also calculated. All analyses were performed using SAS software (version 9.4., SAS Institute, Cary, North Carolina). *P*‐values <.05 were considered significant.

## 3 RESULTS

### 3.1 Animals

Twenty dogs with gliomas were included in the study. The median age of dogs with gliomas was 7 years (range, 4‐12 years). There were 11 spayed females and 9 neutered males. The median body weight was 26 kg (range, 7‐49 kg). Breeds of dogs with glioma included mixed breeds (n = 5), Boston Terrier (n = 4), Boxer (n = 5), American Staffordshire Terrier (n = 3), and 1 each of the following: Bull Terrier, French Bulldog, and Labrador Retriever. All dogs had rostrotentorial forebrain gliomas, with 13 tumors located on the left side of the brain, and 7 on the right side. Eight gliomas were located in the fronto‐olfactory lobes, 5 in the temporal‐piriform lobes, 4 in the fronto‐parietal lobes, and 3 in the temporal‐occipital lobes.

All dogs with glioma had structural epilepsy, 16/20 displayed focal interictal neurological deficits referable to the neuroanatomic location of their tumor within the brain, and 2/20 displayed multifocal intracranial signs. Histopathological diagnoses included 10 high‐grade oligodendrogliomas, 4 low‐grade oligodendrogliomas, 5 high‐grade astrocytomas, and 1 low‐grade astrocytoma.^25^ All dogs were receiving treatment with prednisone (median dose 0.62 mg/kg/d, range 0.33‐1.3 mg/kg/d) at the time MRI examinations and ICP measurements were obtained, and the median duration of prednisone treatment was 9 days (range, 6‐54 days).

The median age of the 3 control dogs was 4 years (range, 3‐5 years), and the median body weight was 10 kg (range, 9‐12 kg). All control dogs were beagle‐mix type dogs and there were 2 spayed females and 1 neutered male.

### 3.2 Clinical and MRI estimates of ICP

Based on a CCS ≥ 4, ICH was presumed to be present in 13/20 dogs with gliomas (clinically predicted ICH group), and in none of the control dogs. No dogs in this study had abnormalities on fundoscopic examination, so the fundoscopic score variable was excluded from further data analysis. Karnofsky performance scores, MGCS, and CCS scores were significantly different between control and tumor dogs as well as between dogs with clinically predicted ICH and those with clinically predicted normal ICP (Table 2). A total of 14/20 dogs with gliomas were diagnosed with presumptive ICH based on MRI features (MRI‐predicted ICH group), and 8/20 dogs with gliomas were estimated to have ICH based on both CCS and MRI assessments. Overall, agreement among the 3 observers was good when assigning binary outcomes to MRI features of presumptive ICH (Supporting Information Table S1). No differences in KPS, MGCS, and CCS scores were found between the MRI‐predicted ICP groups (Table 2).

**TABLE 2:** Comparison of clinical variables and composite clinical scores between clinically predicted and MRI‐predicted ICP groups

	Mean (SD) group scores			Mean (SD) group scores			Mean (SD) group scores		
Clinical assessment (range)	Control	Tumor	*P*‐value	Clinically predicted normal ICP	Clinically predicted ICH	*P*‐value	MRI‐predictednormal ICP	MRI‐predictedICH	*P*‐value
Fundoscopic score (0–1)	0 (0)	0 (0)	NA	0 (0)	0 (0)	NA	0 (0)	0 (0)	NA
Karnofsky performance score (0–100)	100 (0)	75.3 (15.3)	.01*	92.2 (6.7)	69.6 (15.1)	.0001*	82.5 (13.6)	75.4 (18.6)	.66
Modified Glasgow coma scale (3–18)	18 (0)	16.2 (2.0)	.04*	17.9 (.33)	15.5 (2.0)	.0006*	16.8 (1.6)	16.2 (2.2)	.48
Composite clinical score (0‐26)	0 (0)	4.4 (3.4)	.01*	.9 (.9)	5.7 (3.2)	.0008*	3.1 (2.7)	4.4 (4.0)	.62

Abbreviations: ICH, intracranial hypertension; ICP, intracranial pressure; MRI, magnetic resonance imaging.

Statistically significant: *P < .05.

### 3.3 dICP measurements and their relationships with clinical and MRI estimates of ICP

Direct intracranial pressure values were successfully obtained in all dogs. In 3/23 dogs, punctate hemorrhagic foci were noted on the gyral surface in the transducer insertion region after removal of the dICP transducer, but no other adverse events directly attributable to the intraparenchymal dICP transducer placement or removal were observed. All dogs that underwent dICP monitoring in this study received no specific treatment for ICH other than corticosteroids, and 22/23 dogs survived for ≥30 days after the dICP monitoring procedure. One dog with glioma (case T3; Supporting Information Figure S1) was euthanized because of respiratory failure 24 hours after the dICP and glioma therapeutic interventional procedures.

**FIGURE 2:**
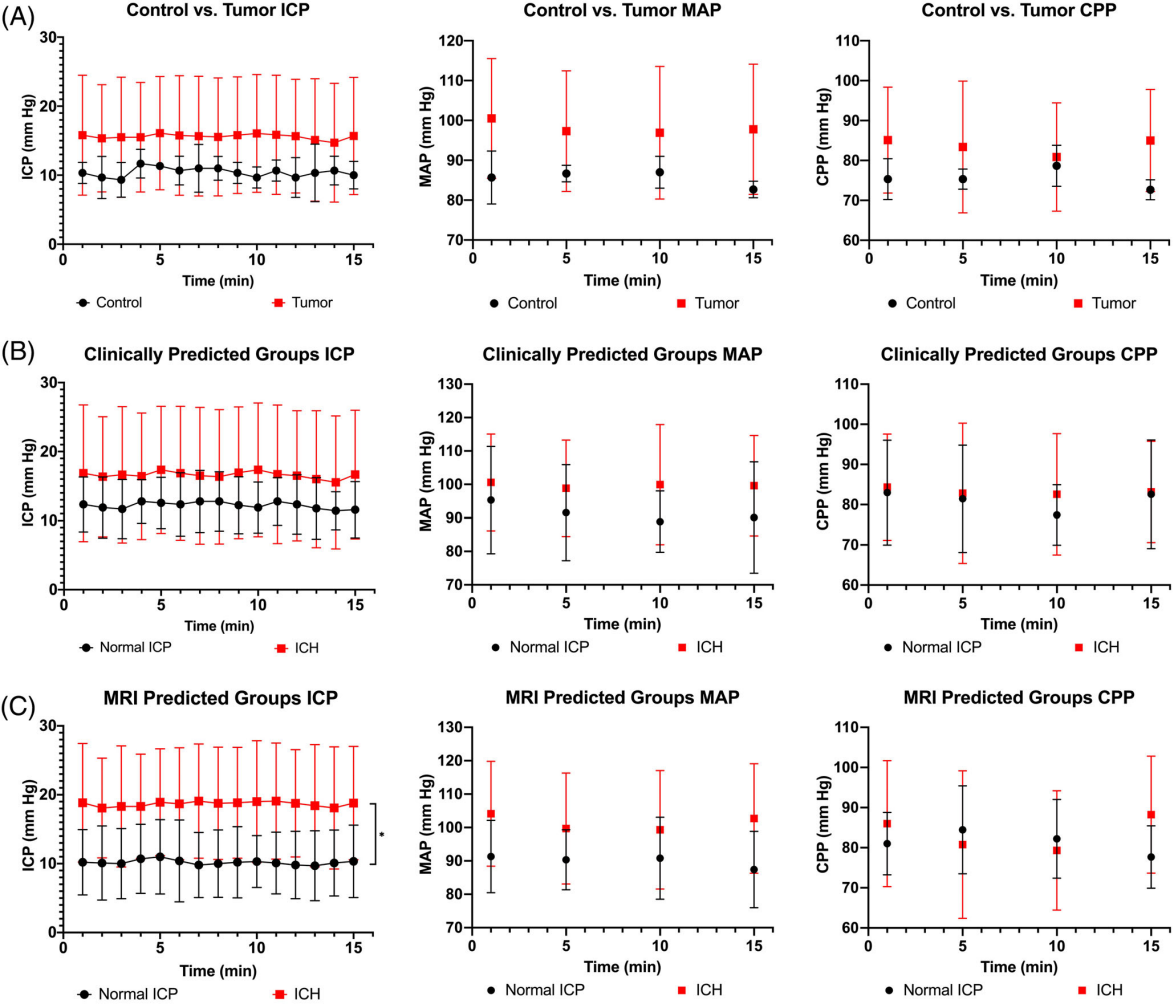
Comparisons of intracranial pressures (ICP; left column), mean arterial pressures (MAP; middle column), and cerebral perfusion pressures (CPP; right column) between control and tumor‐bearing dogs, A, dogs with clinically predicted normal ICP and clinically predicted intracranial hypertension (ICH; B), and dogs with MRI‐predicted normal ICP and magnetic resonance imaging (MRI) predicted ICH, C. The ICP is significantly higher (*P* = .004*) in dogs with MRI‐predicted ICH compared to dogs with MRI‐predicted normal ICP (C, left panel). No significant differences were noted between MAP or CPP in any group

The mean dICP of control dogs was 10.4 ± 2.1 mm Hg and the mean dICP of tumor‐bearing dogs was 15.6 ± 8.3 mm Hg, and these ICP values were not significantly different (*P* = .29; Figure 2A). Among the 20 dogs with gliomas, time‐averaged dICP were <10 mm Hg in 5/20, 10‐15 mm Hg in 5/20, 15‐20 mm Hg in 6/20, 20‐25 mm Hg in 2/20, and 30‐35 mm Hg in 2/20 (Supporting Information Figure S1). Direct intracranial pressure measurements were also not significantly different (*P* = .19) between dogs with clinically predicted normal ICP or clinically predicted ICH over time (Figure 2B). Dogs with MRI‐predicted ICH had significantly higher (*P* = .004) dICP than dogs with MRI‐predicted normal ICP over time (Figure 2C). No significant differences were noted in MAP or CPP values (Figure 2) between control and tumor‐bearing dogs (MAP, *P* = .14; CPP, *P* = .30), dogs with clinically predicted normal ICP and clinically predicted ICH (MAP, *P* = .16; CPP, *P* = .58), or dogs with MRI‐predicted normal ICP and MRI‐predicted ICH (MAP, *P* = .06; CPP, *P* = .80).

Qualitative MRI features that were significantly more likely to be present in dogs classified as having MRI‐predicted ICH compared with those classified as having MRI‐predicted normal ICP included transtentorial, foramen magnum, or subfalcine herniations, caudal displacement of the lamina quadrigemina, 3rd ventricular compression, perilesional edema, and falx shift (Table 3). Quantitatively, dogs with MRI‐predicted ICH also had significantly higher T2W lesion volumes, T2W lesion volume : brain volume ratios, and higher ONSD : body weight ratios when compared to dogs with MRI‐predicted normal ICP (Table 4).

**TABLE 3:** Qualitative MRI feature comparisons between MRI‐predicted ICP groups

MRI feature		MRI‐predicted ICP group; frequency (%)		*P*‐value
		Predicted normal ICP	Predicted ICH	
Sulci effacement	Absent	3 (13%)	0 (0%)	.07
	Present	7 (30%)	13 (57%)	
Transtentorial herniation	Absent	10 (43%)	6 (26%)	.008*
	Present	0 (0%)	7 (30%)	
Foramen magnum herniation	Absent	10 (43%)	8 (34%)	.04*
	Present	0 (0%)	5 (22%)	
Subfalcine herniation	Absent	10 (43%)	8 (34%)	.04*
	Present	0 (0%)	5 (22%)	
Lamina quadrigemina position	Rostral	10 (43%)	5 (22%)	.002*
	Caudal	0 (0%)	8 (34%)	
Perilesional edema	Absent	6 (26%)	0 (0%)	.002*
	Present	4 (17%)	13 (57%)	
Third ventricular compression	Absent	8 (34%)	2 (9%)	.003*
	Present	2 (9%)	11 (48%)	
Fourth ventricular compression	Absent	10 (43%)	10 (43%)	.22
	Present	0 (0%)	3 (13%)	
Falx shift	Absent	9 (39%)	1 (4%)	.0001*
	Present	1 (4%)	12 (52%)	

Abbreviations: ICH, intracranial hypertension; ICP, intracranial pressure; MRI, magnetic resonance imaging.

Statistically significant: *P < .05.

**TABLE 4:** Quantitative MRI feature comparisons between MRI‐predicted ICP groups

MRI feature	MRI‐predicted ICP group	Mean (SD)	*P*‐value
T2W lesion volume (cm^3^)	Predicted normal ICP	1.45 (1.2)	.0004*
	Predicted ICH	5.71 (3.03)	
T2 brain volume (cm^3^)	Predicted normal ICP	77.32 (13.12)	.28
	Predicted ICH	84.78 (18.08)	
T2W lesion volume : brain volume	Predicted normal ICP	0.02 (0.01)	.0004*
	Predicted ICH	0.06 (0.03)	
Right optic nerve sheath diameter : BW	Predicted normal ICP	0.24 (0.18)	.01*
	Predicted ICH	0.33 (0.26)	
Left optic nerve sheath diameter : BW	Predicted normal ICP	0.22 (0.17)	.04*
	Predicted ICH	0.35 (0.21)	

Abbreviations: BW, body weight; ICH, intracranial hypertension; ICP, intracranial pressure; MRI, magnetic resonance imaging.

Statistically significant: *P < .05.

Receiver operating characteristic curve analysis indicated that a dICP defined ICH threshold of 15 mm Hg demonstrated the highest combined sensitivities and specificities for both clinical (AUC = 0.58; 95% CI, 0.38‐0.79) and MRI (AUC = 0.80; 95% CI, 0.63‐0.96) predictions to differentiate between dogs with and without ICH. At a dICP threshold of 15 mm Hg, the sensitivity of the CCS for predicting ICH was 70% and the specificity 46%, and the sensitivity for MRI for predicting ICH was 90% and the specificity 69%.

## 4 DISCUSSION

Obtaining acute, intraoperative direct intraparenchymal ICP measurements is feasible using the described transducer and technique in dogs with forebrain tumors. The dICP values we obtained in control and glioma dogs approximated baseline values reported in other studies in anesthetized healthy and tumor‐bearing dogs.^5^, ^10^ In this study population, dICP values were not significantly different between control and tumor‐bearing dogs.

Our inability to detect a difference in ICP between control and tumor‐bearing dogs may have resulted from the limited sample size of our control group, or from our finding that only 50% (10/20) of the glioma dogs in our population had dICP above the threshold we considered potentially compatible with ICH (≥15 mm Hg), based on dICP values in neurologically normal anesthetized dogs obtained in this and another study.^10^ Additionally, only 4/10 glioma dogs in our study with ICH had dICP ≥20 mm Hg. Established definitions of ICH based on absolute dICP values do not currently exist for dogs with naturally occurring intracranial disease, and likely will differ based on patient age, breed anatomical variability, underlying brain disease etiology, anesthetic protocol, and head or body position during dICP recordings.^8^, ^9^, ^10^, ^11^ Although 1 study investigating dICP values in dogs with brain tumors defined ICH as >13 mm Hg,^5^ ICH has been previously defined as sustained dICP values ≥20 mm Hg, as pressures above this threshold have been shown to be detrimental to cerebral perfusion or clinical outcome in dogs with experimentally induced ICH, brain tumors, and spontaneous TBI.^2^, ^4^, ^26^

This study attempts to validate clinical and MRI surrogates of presumptive ICH with dICP measurements in dogs with naturally occurring brain disease. The CCS system used in this study was not able to reliably presumptively discriminate dogs with normal ICP from those with ICH based on the dICP results from those 2 groups, even though dogs with clinically predicted ICH had significantly worse (ie, indicative of more severe clinical dysfunction) CCS, KPS, and MGCS scores. Although the severity of neurologic dysfunction has been demonstrated to have prognostic importance in dogs undergoing treatment for brain tumors, there are no clinical assessment instruments that have been validated as quantitative clinical disease burden surrogates in dogs with brain tumors.^27^, ^28^ We chose to construct the CCS from fundoscopic, KPS, and MGCS scores as these have been used as indicators of ICH in dogs with brain tumors, or have prognostic value in the assessment of dogs with cancer or TBI.^12^, ^21^, ^22^ Our CCS results highlight the need to develop specific clinical scoring systems for dogs that can be validated against clinical or pathobiological outcomes relevant to the disease of interest. Our study also indicates that papilledema is rarely observed in dogs with intracranial gliomas, even in the face of confirmed ICH, and further supports prior investigations reporting that 40% to 75% of dogs with imaging evidence of brain herniations have no clinical signs specifically referable to those herniations.^29^, ^30^

Our results corroborate that a substantial number of dogs with intracranial mass lesions causing mild to moderate clinical neurological dysfunction have baseline dICP values within reference ranges.^2^, ^5^ Given that the MAP and CPP values of dogs in this study were also within putative ranges consistent with preserved cerebral autoregulatory functions and not different between control and tumor‐bearing groups, our data suggest that even those dogs with an elevated ICP were likely in the compensatory phases of ICH.^1^ This supports that multiple pathophysiological mechanisms, such as compression or invasion of neural tissue, neuroinflammation, and ICH, all likely contribute to the neurological dysfunction observed in dogs with focal brain tumors.

Direct intracranial pressures were significantly higher in the MRI‐predicted ICH group, and dogs with MRI‐predicted ICH were significantly more likely to have evidence of multiple anatomical structural shifts on MRI examinations. Magnetic resonance imaging features previously identified as indirect indicators of ICH such as transtentorial, foramen magnum, or subfalcine herniations, caudal displacement of the lamina quadrigemina, 3rd ventricular compression, perilesional edema, and falx shift were also significantly more likely to be present in dogs with MRI‐predicted ICH in this study.^12^, ^13^, ^14^ We also observed that dogs with MRI‐predicted ICH had significantly larger T2W lesions and T2W lesion volume : brain volume ratios, which is intuitive given the inherent associations of these lesion burden indicators with features of mass effect.^31^ In this study, MRI evidence of brain herniations were notably absent in all dogs in the MRI‐predicted normal ICP group, which highlights the negative predictive value of the absence of these imaging findings when estimating ICP. Prior studies estimating ICP in dogs based on MRI findings assigned dogs to presumed ICH groups when ≥2 MRI features examined in this study were present.^12^, ^13^ If we had applied similar scoring criteria, 19/20 dogs with gliomas would have been assigned to the MRI‐predicted ICH group, and further contributed to the observed Type I error.

Quantification of the ONSD on MRI is a reliable indicator of ICH in humans, and previous studies in dogs have demonstrated relationships between larger ONSD measurements and indirect estimates of ICH and dICP in dogs.^13^, ^17^, ^32^ We also found that the ONSD : body weight ratios were significantly higher in dogs with MRI‐predicted ICH, and our ONSD : body weight ratios were similar to previously reported values in dogs with intracranial disease.^13^ To further investigate the clinical utility of ONSD: body weight ratios as indicators of ICH, future studies should include comparisons of dICP measurements with MRI quantification of ONSD, along with other noninvasive estimates of ICP, such as ultrasonographic measurements of ONSD or TCD.^13^, ^14^, ^16^ Identification of a readily obtained, noninvasive bedside indirect surrogate of ICP could greatly facilitate the management of ICH in the acute clinical setting.

Sulci effacement and 4th ventricular compression were the only MRI features examined in this study that were not significantly more likely to be noted in the MRI‐predicted ICH group. The discriminatory value of sulci effacement was limited in this population, as all of the glioma dogs in this study displayed this MRI abnormality, which is not surprising given that our population consisted exclusively of dogs with rostrotentorial intra‐axial tumors.^31^ Based on the findings in this and other studies, sulci effacement will be observed in many dogs with rostrotentorial tumors but should not be used to infer the presence of ICH on its own. It is likely that our study did not find statistical significance between 4th ventricular compression and ICH because of that fact that all of the animals in our study had focal forebrain tumors, with only 3/23 dogs demonstrating 4th ventricular compression. It remains possible that sulci effacement and 4th ventricular compression may be more sensitive indicators of ICH in dogs with other etiologies of intracranial disease, or with lesions arising in the caudal fossa, respectively.^12^

Given the sensitivity and specificity results of the clinical estimates of ICP observed in this study, we cannot recommend presumptively treating dogs with mild to moderate neurological dysfunction for ICH based on clinical evaluations. Our findings do indicate that administration of mannitol or hypertonic saline solutions might be indicated in dogs with multiple MRI features of ICH, although this will result in indiscriminate treatment of ICH in at least 30% of dogs with brain tumors. Thus, our study also highlights the necessity to interpret the significance of MRI findings in appropriate clinical context. Although it was not an objective of our study to assess the prognostic impact of dICP, 22/23 of the dogs in this study survived for ≥30 days after the dICP measurements, and the tumor‐bearing dog that died in the acute perioperative period had a normal ICP. The presence of ICH upon recovery form anesthesia after craniotomy was a significant risk factor for acute perioperative mortality in dogs with intracranial tumors, and we observed similar baseline dICP, MAP, and CPP values in our tumor‐bearing dogs.^5^ Additional dICP studies are needed to further define the impact of ICH and its treatment on clinically relevant outcomes such as anesthetic recovery, perioperative adverse events, neurological status, and survival.^4^, ^5^

As our study design included a relatively homogenous clinical population, our results may not be representative of dogs with untreated brain tumors, relevant in dogs with caudal fossa lesions, or generalizable to dogs with anomalous, inflammatory, metabolic, traumatic, or vascular diseases that can cause ICH. Whereas our sample of tumor dogs may also not be representative of the entire population of dogs with brain tumors, it is representative of those dogs that are generally considered reasonable clinical candidates for anesthesia and surgical intervention, and would therefore be candidates for dICP monitoring.^5^, ^28^ Additionally, studying this group of dogs was advantageous in that the anesthetic protocol, head and body position, surgical approach, limited craniectomy size, and dural puncture were standardized, as all of these variables are known to potentially influence ICP.^1^, ^2^, ^9^, ^10^, ^11^

## 5 CONCLUSIONS

Obtaining dICP measurements in dogs with forebrain tumors is feasible using the described microsensor and technique. In tumor‐bearing dogs with mild to moderate neurological dysfunction, MRI features including brain herniations, mass effect, and ONSD size aid identification of dogs with ICH. Clinical estimation of ICP did not reliably discriminate between dogs with and without ICH in this population.

## CONFLICT OF INTEREST DECLARATION

Authors declare no conflict of interest.

## OFF‐LABEL ANTIMICROBIAL DECLARATION

Authors declare no off‐label use of antimicrobials.

## INSTITUTIONAL ANIMAL CARE AND USE COMMITTEE (IACUC) OR OTHER APPROVAL DECLARATION

The study was conducted in accordance with the guidelines of the Virginia Tech Institutional Animal Care and Use Committee (protocols 17‐203, 15‐221, 14‐235, 12‐014, and 08‐218).

## HUMAN ETHICS APPROVAL DECLARATION

Authors declare human ethics approval was not needed for this study.

## Supporting information


**Figure S1** Mean (±SD) intracranial pressures obtained from individual control (C) and tumor‐bearing (T) dogs.

**Table S1** Interobserver agreement for quantitative MRI features of ICH.
