# Supplementary figures and images for: Comparison of direct measurement of intracranial pressures and presumptive clinical and magnetic resonance imaging indicators of intracranial hypertension in dogs with brain tumors
Source: J Vet Intern Med. 2020 May 16;34(4):1514–23. doi: 10.1111/jvim.15802 (PMC7379039; doi:10.1111/jvim.15802)

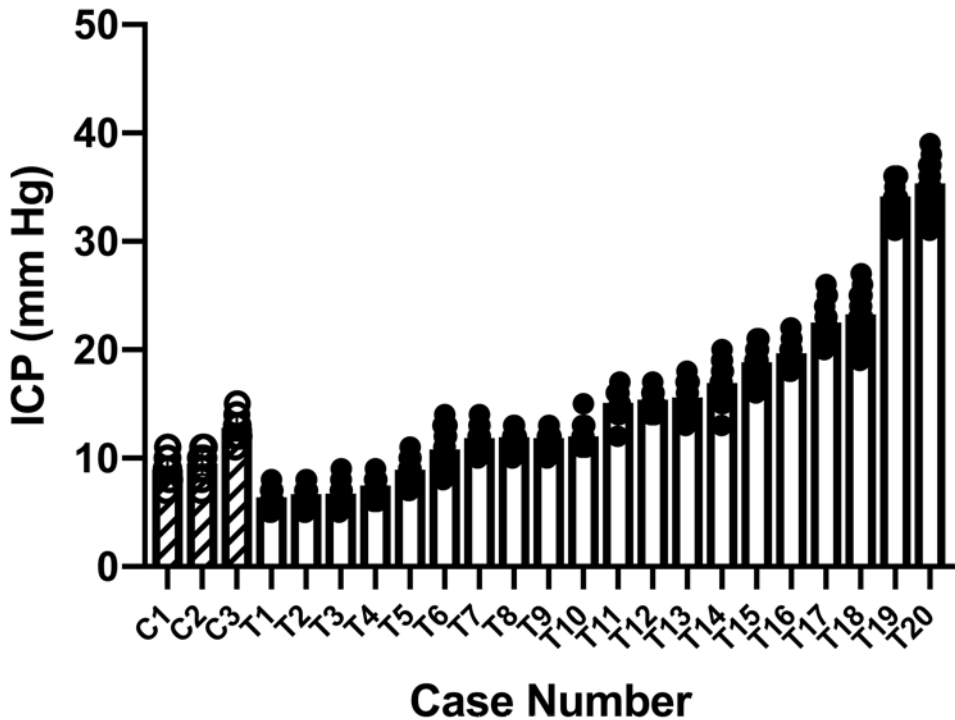

Supplement: Supplementary file 1 — Figure S1 Mean (±SD) intracranial pressures obtained from individual control (C) and tumor‐bearing (T) dogs. [file JVIM-34-1514-s001.pdf]
