# Supplementary material for: Comparison of direct measurement of intracranial pressures and presumptive clinical and magnetic resonance imaging indicators of intracranial hypertension in dogs with brain tumors
Source: J Vet Intern Med. 2020 May 16;34(4):1514–23. doi: 10.1111/jvim.15802 (PMC7379039; doi:10.1111/jvim.15802)
Supplement: Supplementary file 2 — Table S1 Interobserver agreement for quantitative MRI features of ICH. [file JVIM-34-1514-s002.pdf]

Supplemental Table S1. Interobserver agreement for quantitative MRI features of ICH.

	**Percent Agreement**	**Kappa statistics**			**Intra-class correlation coefficient**
**Measure**		**Observers 1 and 2**	**Observers 1 and 3**	**Observers 2 and 3**	
Sulci effacement	0.97	0.83	1.00	0.83	0.89
Transtentorial herniation	1.00	1.00	1.00	1.00	1.00
Foramen magnum herniation	0.97	0.88	0.88	1.00	0.92
Subfalcine herniation	1.00	1.00	1.00	1.00	1.00
Lamina quadrigemina position	0.88	0.70	0.70	0.79	0.74
Perilesional edema	1.00	1.00	1.00	1.00	1.00
Third ventricular compression	0.97	0.91	0.91	1.00	0.94
Fourth ventricular compression	1.00	1.00	1.00	1.00	1.00
Falx shift	1.00	1.00	1.00	1.00	1.00
MRI predicted ICH	1.00	1.00	1.00	1.00	1.00
